# SSTA-ResT: Soft Spatiotemporal Attention ResNet Transformer for Argentine Sign Language Recognition

**DOI:** 10.3390/s25175543

**Published:** 2025-09-05

**Authors:** Xianru Liu, Zeru Zhou, E Xia, Xin Yin

**Affiliations:** 1 School of Automation, Central South University, Changsha 410083, China; liuxianru@csu.edu.cn (X.L.); zzrcsu@csu.edu.cn (Z.Z.);; 2 Information and Network Center, Central South University, Changsha 410083, China

**Keywords:** Argentine Sign Language recognition, ResNet, spatiotemporal attention, Transformer

## Abstract

Sign language recognition technology serves as a crucial bridge, fostering meaningful connections between deaf individuals and hearing individuals. This technological innovation plays a substantial role in promoting social inclusivity. Conventional sign language recognition methodologies that rely on static images are inadequate for capturing the dynamic characteristics and temporal information inherent in sign language. This limitation restricts their practical applicability in real-world scenarios. The proposed framework, called SSTA-ResT, integrates ResNet, soft spatiotemporal attention, and Transformer encoders to achieve this objective. The framework utilizes ResNet to extract robust spatial feature representations, employs the lightweight SSTA module for dual-path complementary representation enhancement to strengthen spatiotemporal associations, and leverages the Transformer encoder to capture long-range temporal dependencies. Experimental results on the LSA64 Argentine Sign Language (ASL) dataset demonstrate that the proposed method achieves an accuracy of 96.25%, a precision of 97.18%, and an F1 score of 0.9671. These results surpass the performance of existing methods across all metrics while maintaining a relatively low model parameter count of 11.66 M. This demonstrates the framework’s effectiveness and practicality for sign language video recognition tasks.

## 1. Introduction

Sign language is the primary medium of communication for deaf and hard-of-hearing individuals, facilitating the conveyance of rich cultural and emotional expressions. Globally, approximately 72 million deaf individuals reside in low- and middle-income countries, accounting for over 80% of the global deaf population. These individuals utilize over 300 distinct sign languages [1]. Sign language is defined as a distinct linguistic system that utilizes manual gestures, facial expressions, and bodily movements to convey thoughts and emotions. The components of sign language encompass hand shapes, the trajectory of gestures, the direction of gestures, the velocity of gestures, facial expressions, and body postures. The elements of sign language are integrated to create a unique and expressive style [2,3,4]. For instance, the “spatial” characteristic of sign language enables gestures to express different meanings in three-dimensional space, while the “simultaneity” characteristic facilitates the synchronous conveyance of multiple pieces of information through gestures and facial expressions. The utilization of sign language transcends mere daily communication within the deaf community; it serves as a pivotal medium for the articulation of their cultural identity and facilitation of social participation. However, the general population’s concerns regarding the significant time and economic demands of acquiring sign language, coupled with their overall indifference toward it, have led to mounting challenges in facilitating effective communication between deaf individuals and the general population. Consequently, the development of efficient sign language recognition technology is imperative to reduce the economic costs of communication between the deaf community and the general population. This technology is crucial for promoting communication between the deaf community and other groups, improving the quality of education for deaf people, and advancing social integration.

### 1.1. Improvements in Recognition Algorithms

In early algorithm development, researchers employed various machine learning methods, such as Hidden Markov Models (HMMs) [5] and support vector machines (SVMs) [6], among others. However, these methods have always relied on the experience of designers, and the time series modeling process is cumbersome and difficult to improve. In recent years, researchers have adopted deep neural networks for sign language video recognition. Convolutional neural networks (CNNs) can automatically extract spatial features from images or video frames, such as hand shapes, hand positions, and motion trajectories [7], while recurrent neural networks (RNNs) can extract temporal features of sign language movements [8]. These applications have significantly improved the accuracy and speed of sign language recognition.

Nevertheless, despite the efficacy of these methodologies for sign language recognition, several issues persist. As the amount of data increases and the number of network layers grows, neural networks are susceptible to stability issues such as gradient explosion and gradient vanishing [9]. Furthermore, time series processing for sign language recognition necessitates the capture of both short-term sequences and long-term dependencies to ensure that the model can accurately identify the correlations between complete actions [10].

Numerous models have demonstrated a high recognition accuracy and exceptional performance in sign language video applications. Nonetheless, in practical scenarios, the devices employed for sign language recognition, such as smartphones, possess relatively limited graphical processing capabilities. Consequently, model design must strike a balance between achieving a high accuracy and minimizing computational costs, thereby reducing computational demands to facilitate deployment on edge-portable devices and effectively address the practical needs of individuals with hearing impairments.

### 1.2. Format of Sign Language Recognition Datasets

**Figure 1:**
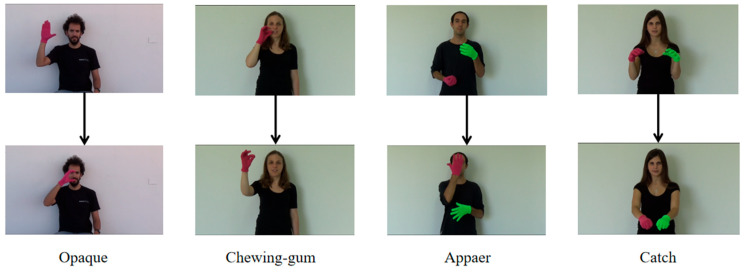
Demonstration of some words in the dataset, with each word expressed through hand movements. (All participants in the photos were wearing red and green fluorescent gloves, which will be detailed in Section 3.2 later).

Early data collection for sign language recognition, such as American Sign Language (ASL), was primarily constrained to static images [11]. While this approach made significant contributions to the field of sign language recognition, image-based sign language datasets still have significant limitations. Many signs cannot be represented statically and must rely on hand movements to be completed. Consequently, sign language recognition through video has garnered mounting interest in recent years. In comparison with static image-based sign language recognition, video-based sign language recognition can capture the dynamic changes and continuity of gestures, thereby providing richer contextual information and improving recognition accuracy and robustness [12]. Furthermore, the capacity of video sign language recognition to process continuous sign language streams aligns with the natural expression patterns of sign language, thereby overcoming the limitations of static image methods in handling continuous gestures [13]. By modeling the spatiotemporal characteristics of sign language videos, the recognition of sign language videos can be enhanced through comprehension of the grammatical and semantic structures of sign language, thereby improving the performance of recognition systems [14]. As visualized in Figure 1, the LSA64 dataset contains dynamic gesture representations where each sign language word is expressed through sequential hand movements. This demonstrates the spatiotemporal complexity requiring frame-level modeling in our SSTA-ResT framework.

In this study, we consider the optimization of ResNet’s residual network for issues related to overfitting and the Transformer’s efficient feature extraction capabilities and lower computational costs in long-term sequence processing, and combine them with low-parameter soft spatiotemporal attention to propose a more cost-effective video-based sign language recognition framework. The proposed framework is predicated on ResNet and Transformer, with ResNet being utilized for the extraction of features, spatiotemporal attention modules being integrated for the purpose of emphasizing hand movements, and the Transformer encoder being employed for the extraction of global temporal dependencies. Ultimately, a fully connected layer is employed for classification. The primary contributions of this study can be enumerated as follows:

- We hereby propose a novel fusion network, SSTA-ResT, for action video classification and recognition. The network utilizes a residual network to extract spatial features, a spatiotemporal attention module to capture the relationship between temporal and spatial features, and a Transformer encoder to extract long-term temporal dependencies. This approach is demonstrated to enhance classification performance on the dataset.
- We propose a lightweight convolutional soft attention mechanism, SSTA (Soft Spati-Temporal Attention), which is specifically designed for video sequence feature enhancement. This mechanism enhances spatiotemporal correlations by generating dual complementary representations.
- The efficacy of our word-level sign-language-based motion recognition approach is demonstrated to exceed that of alternative methodologies. The findings of our empirical investigation indicate that the model exhibits substantial enhancements in recognition performance in comparison with the outcomes of prior studies.

## 2. Literature Review

Sign language recognition (SLR) projects have been identified as a crucial conduit for fostering communication and collaboration between deaf and hearing communities. In recent years, driven by advances in artificial intelligence technology, numerous research projects have been conducted around the world, achieving significant progress. Research on SLR involves multiple interdisciplinary fields, including but not limited to sensor technology, machine learning, and deep learning techniques such as convolutional neural networks (CNNs) and recurrent neural networks (RNNs).

The development of sensor technology has been instrumental in establishing the foundation for sign language recognition systems, enabling the acquisition of gesture data. The evolution of this technology has led to a substantial augmentation in the scope and precision of data collection. Conventionally, sign language recognition relied on sophisticated visual capture devices. However, recent advancements in sensor technology have led to the emergence of wearable and non-contact sensors, which have begun to play a significant role [15]. Liu et al. proposed a flexible triboelectric nanogenerator sensor integrated into a smart glove for the purpose of monitoring the unique voltage signal patterns of sign language letters and Chinese characters [16]. Song et al. introduced a wearable wrist sensor based on Multi-Walled Carbon Nanotube/Cotton Fabric Material, which featured a high sensitivity, fast response time, and excellent stability, enabling gesture recognition through a Convolutional Neural Network–Bidirectional Long Short-Term Memory Network model [17]. Despite the advances in sensor technology, which provide a multitude of data sources for sign language recognition, the field continues to face significant challenges. For instance, factors such as the comfort level experienced by individuals when wearing sensors, the presence of noise and drift in sensor data, and the intricacy of data synchronization and fusion between disparate sensors must be taken into consideration.

Machine learning constitutes the primary driving force in the field of sign language recognition. The capacity for systems to discern and comprehend the intended meaning of gestures is predicated on the analysis of patterns inherent in sign language data. The proliferation of data and advancements in computational capabilities have led to the widespread adoption of various conventional machine learning algorithms for sign language recognition. Vanita Jain et al. employed support vector machines (SVMs) and convolutional neural networks (CNNs) for the purpose of American Sign Language recognition [18]. Novianty and Azmi employed Principal Component Analysis (PCA) and support vector machine (SVM) for the purpose of recognizing Indonesian Sign Language [19]. Chang and Pengwu proposed a method for gesture recognition that integrated curvature scale space (CSS) and a Hidden Markov Model (HMM). This approach utilized CSS descriptors to characterize hand shapes and the HMM for sign language recognition [20]. While these methods have been shown to demonstrate a remarkable performance on certain gestures, sign language recognition that relies primarily on traditional machine learning algorithms still faces challenges such as an overall low accuracy and excessive computational time requirements.

In recent years, convolutional neural networks (CNNs) and recurrent neural networks (RNNs) have become the mainstream methods in sign language recognition due to their outstanding performance in image recognition, feature extraction, and temporal modeling [21,22]. Ying Ma et al. proposed a novel dual-stream hybrid CNN model for processing the nonlinear transformation of features during convolution operations for American Sign Language (ASL) [23]. Teran-Quezada et al. developed a system using an LSTM model to convert Panamanian Sign Language into Spanish text, highlighting the effectiveness of LSTM in processing sequential data and achieving the continuous capture of sign language translation patterns [24]. Ariesta et al. achieved commendable outcomes in the realm of Indonesian Sign Language recognition through the synergistic integration of the strengths inherent in 3D CNN and bidirectional recurrent neural networks (Bi-RNNs) [25]. MuthuMariappan and Gomathi proposed a hybrid ConvNet-LSTM network for dynamic gesture recognition in Indian Sign Language. This network was capable of extracting spatial and temporal features from real-time video to effectively recognize sign language vocabulary [26].

In the domain of Argentine Sign Language (ASL) research, Franco et al. proposed a general probabilistic model for symbol classification, achieving a commendable performance on the Argentine Sign Language (ASL) dataset using the bag-of-words method [27]. Geovane proposed a 3D-CNN architecture and achieved a high accuracy in dataset testing [28]. Huang and Chouvatut proposed a ResNet+LSTM architecture for Argentine Sign Language recognition, achieving significant breakthroughs compared to traditional machine learning models and convolutional neural networks [29].

In the field of temporal modeling, the rise of Transformers has further augmented models’ capacity to capture long-range dependencies, with numerous researchers applying them to image recognition tasks. Tan et al. employed a ViT with a gesture recognition attention mechanism in the field of gesture recognition and achieved good results on relevant datasets [30]. Alnabih and Maghari’s research explored the application of Vision Transformers in recognizing static Arabic Sign Language letters. Their findings indicated that, in comparison to conventional CNN methods, ViTs may possess an advantage in capturing global contextual information [31].

## 3. Methods and Materials

### 3.1. Overview

**Figure 2:**
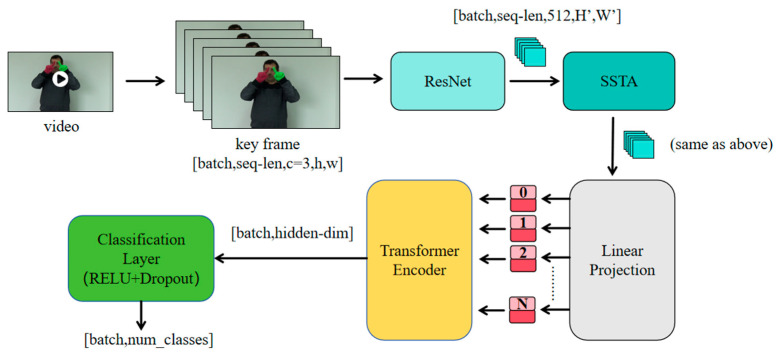
The entire framework processing flow. (Blue path: ResNet spatial feature extraction; Green blocks: SSTA’s dual-path spatiotemporal enhancement; Orange modules: Transformer-based temporal encoding, and Purple layer: final classification. The color-coded components highlight our hierarchical feature fusion strategy.).

Gradient overfitting and explosion have long plagued neural network training, leading to a poor robustness and performance in sign language recognition tasks. Capturing temporal video sequences and integrating them with spatial features, such as hand poses, is essential for model improvement. To tackle these challenges, we propose the SSTA-ResT framework, combining the ResNet and Transformer architectures for video-based sign language recognition. Videos are first preprocessed into a spatiotemporal sequence of 16 key frames and fed into the framework. The process begins with ResNet’s residual network extracting spatial features from sign language gestures. Next, the spatiotemporal attention (SSTA) module generates two complementary representations to enhance video sequences. The Transformer encoder then captures long-range temporal dependencies. Finally, a fully connected layer performs classification to output predictions. The overall architecture and processing flow of SSTA-ResT are illustrated in Figure 2, offering a visual summary of ResNet’s spatial extraction, SSTA’s spatiotemporal enhancement, and the Transformer’s temporal modeling integration.

For a detailed algorithmic representation, the complete process pseudo-code and annotations are provided in Algorithm 1, outlining the step-by-step implementation from input preprocessing to classification.
**Algorithm 1:** SSTA-ResT:Spatio-Temporal attention ResNet transformer
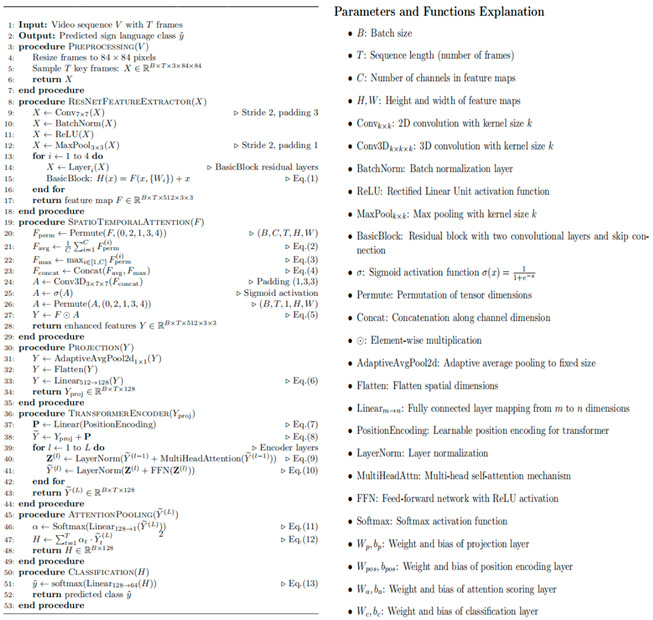


### 3.2. Dataset Preparation and Preprocessing

This study used the LSA64 Argentine Sign Language dataset [32]. The dataset consists of 3200 video recordings of sign language actions, covering 64 commonly used vocabulary items in Argentine Sign Language (LSA). Each category of sign language demonstrations was performed by 10 right-handed participants who were not sign language experts, with each participant performing each demonstration five times. The duration of each demonstration ranged from 0.5 to 2 s. Of the total, 22 vocabulary items were performed using both hands, while 42 were performed using only the right hand. Participants wore black clothing and performed the gestures while wearing fluorescent gloves against a white background. This setup effectively simplified hand segmentation and eliminated the influence of skin tone variations while maintaining the challenge of hand shape recognition. The videos in the dataset were recorded under the following two distinct lighting conditions: natural light and artificial light, thereby preserving lighting variations.

**Figure 3:**
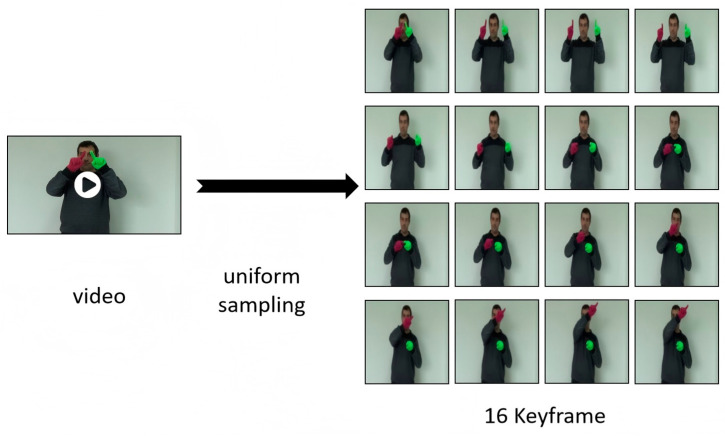
This figure shows the video processing process.

In video recognition, which involves analyzing long-term spatiotemporal sequences, videos are not typically used as raw input directly. In this experiment, the study first reduced the size of each video to 84 × 84 pixels to minimize computational costs while preserving important features. Subsequently, each video was uniformly sampled to extract 16 key frames, forming a 16 × 84 × 84 spatiotemporal sequence. This approach is critical for sign language recognition, as it tests the model’s ability to understand the linguistic content of the signs rather than memorizing the specific motion patterns of individual performers. All sign language videos from two participants were designated as the test set, containing 640 samples (2 participants × 64 signs × 5 repetitions). The videos from the remaining eight participants constituted the training set, containing 2560 samples (8 participants × 64 signs × 5 repetitions). This split strategy effectively prevented data leakage and provided a robust evaluation of the model’s performance on unseen individuals, which is essential for real-world deployment. In Figure 3, we visually demonstrate how a video is divided into 16 keyframes.

**Figure 4:**
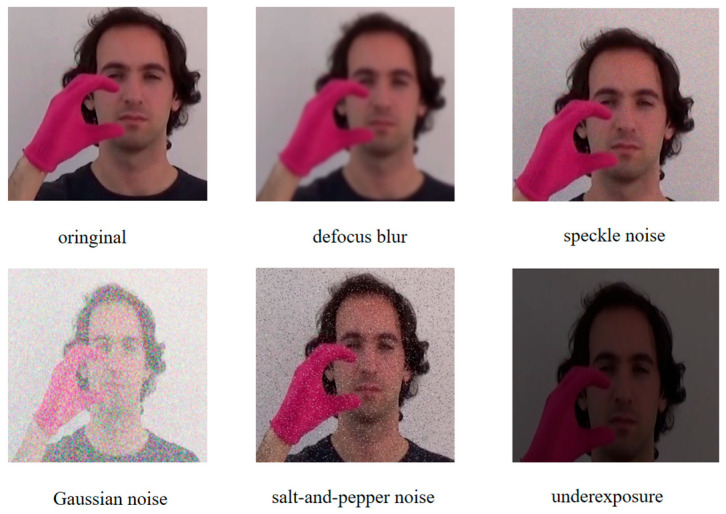
This figure shows the effect of data augmentation.

Our data augmentation pipeline incorporated five stochastic transformations—underexposure, defocus blur, Gaussian noise, salt-and-pepper noise, and speckle noise—applied at the frame level to simulate real-world visual variations and enhance model robustness for sign language recognition under challenging conditions. Figure 4 validates our augmentation pipeline: Original frames (left) transform under five noise types (right). Red borders denote underexposure, yellow defocus blur, cyan Gaussian noise, magenta salt-and-pepper noise, and blue speckle noise—critical for simulating real-world degradation.

### 3.3. ResNet Feature Extraction Module

In recent years, deep learning has demonstrated a remarkable proficiency in complex tasks such as image recognition and natural language processing, largely attributable to the advanced modeling capabilities of deep neural networks. However, as the number of network layers increases, the model’s performance does not continue to improve; rather, a degradation issue may emerge, characterized by an increase in the training error rate rather than a decrease. This phenomenon does not stem from overfitting; rather, it is attributable to the instability of the gradient descent algorithm in excessively deep networks. In most cases, convergence becomes more difficult, and in extreme cases, gradient vanishing or explosion may occur. This degradation issue imposes limitations on the design and implementation of deep networks, impeding the capacity to extract potentially more sophisticated feature representations from deeper layers.

ResNet, or residual networks, was proposed by He et al. in 2015. It is an innovative solution to the degradation problem in deep neural networks [33]. The fundamental premise of this approach entails the implementation of a “residual learning” strategy, which utilizes shortcut connections, also referred to as “jump connections”, to facilitate the transmission of specific information through one or more layers without undergoing the conventional processing path. “Residual blocks” represent a distinct application of “residual learning”. Residual blocks facilitate the network’s learning of residual functions $F(x_{l},W_{l})$, whereby the original mapping $H(x_{l})$ is decomposed into the sum of an identity mapping $h(x_{l})$ and a residual function $F(x_{l},W_{l})$, i.e., as follows:

(1) $$H(x_{l})=h(x_{l})+F(x_{l},W_{l})$$

When the identity mapping is the optimal solution, the residual function only needs to learn to be zero, which greatly simplifies the learning process.

From the perspective of gradient propagation, according to the chain rule, the gradient of the loss function for the input of a certain layer is as follows:

(2) $$\frac{\partial loss}{\partial x_{l}}=\frac{\partial loss}{\partial x_{L}}\cdot \frac{\partial x_{L}}{\partial x_{l}}$$

Due to the presence of residual blocks, $\frac{\partial x_{L}}{\partial x_{l}}$ includes the gradient of the identity mapping (value of 1) and the gradient part of the residual function, i.e.,

(3) $$\frac{\partial x_{L}}{\partial x_{l}}=1+\frac{\partial }{\partial x_{L}}\sum_{i=l}^{L-1}F(x_{i},W_{i})$$

This signifies that the gradient does not undergo a precipitous decay during the process of backpropagation as the number of layers increases. Instead, it maintains a relatively substantial value, thereby ensuring effective updates to the parameters of shallow networks and facilitating the successful training of deep networks. This straightforward yet effective design facilitates ResNet’s attainment of performance breakthroughs across a multitude of tasks, yielding an exceptional performance with diminished model complexity.

The present paper utilizes the benefits offered by residual networks to construct the ResNetFeatureExtractor module, which is employed for the purpose of feature extraction. The module employs an enhanced ResNet-18 architecture as the fundamental feature extractor, thereby effectively addressing the gradient vanishing problem in deep networks through the utilization of residual connections. This approach enables the preservation of low-level visual information in the input features via an identity mapping mechanism. The module commences with a convolution layer that utilizes a 7 × 7 convolution kernel (stride of 2), followed by batch normalization (BatchNorm) and ReLU activation functions. It then initiates the process of initial downsampling through a 3 × 3 max pooling layer, thereby generating an initial feature map comprising 64 channels.

During the feature extraction stage, the module consists of four residual layers (layer 1–layer 4), each composed of BasicBlocks, with each layer containing two residual blocks. Specifically, layer1 maintains the spatial resolution unchanged, while layers 2 to 4 progressively halve the feature map size through convolution operations with a stride of 2, simultaneously increasing the number of channels to 128, 256, and 512, respectively. Each residual block adopts a stacked structure with 3 × 3 convolution kernels and introduces shortcut connections to achieve identity mapping. When the input and output dimensions do not match, the module adjusts the number of channels and downsamples through a 1 × 1 convolution. All convolution layers are followed by batch normalization layers to accelerate training convergence.

**Figure 5:**
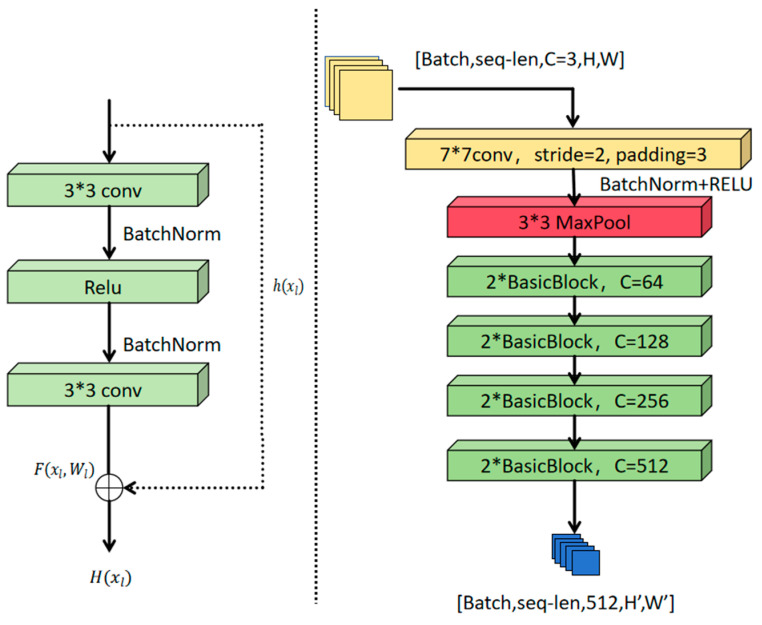
The (**right**) side of the figure shows the architecture of the ResNet feature extraction module, while the (**left**) side shows the structure of the BasicBlock. Yellow refers to the “7*7 conv, stride = 2, padding = 3” convolution layer. Red refers to the “3*3 MaxPool” layer. Green refers to the “BasicBlock” layers.

To further optimize feature expression capabilities, the module uses the Kaiming initialization method to initialize the weights of the convolutional layers [34] and sets the scaling parameter γ of the batch normalization layer to a unit value and the bias parameter β to zero. Ultimately, the module outputs a deep feature map with 512 channels, whose spatial resolution is reduced to 1/32 of the input image, effectively aggregating multi-scale spatiotemporal semantic information to provide high-discriminative input features for subsequent spatiotemporal attention mechanisms. Figure 5 details ResNet’s architecture: Gray arrows indicate identity mappings in BasicBlocks (left), while color-coded layers (right) show channel progression (blue: 64, green: 128, orange: 256, red: 512 channels). The asterisk (*) denotes dimension-matching 1 × 1 convolutions.

### 3.4. Soft Spatiotemporal Attention Mechanism (SSTA)

Traditional convolutional neural networks frequently encounter difficulties in effectively capturing the complex spatiotemporal dependencies that are inherent to sign language movements. As a visual language that is contingent on temporal and spatial information, the semantic expression of sign language is influenced by the spatial form of individual gestures and the evolutionary process of continuous action sequences over time. In order to address this fundamental issue, the present paper puts forth a novel spatiotemporal soft attention module (Soft Spati-Temporal Attention), which is capable of adaptively learning the importance weights of different spatiotemporal regions in video sequences. This, in turn, enhances the model’s ability to represent key sign language features.

The key idea of this module is to model the correlation between temporal and spatial dimensions through three-dimensional (3D) convolution operations. Given the extracted space–time feature tensor $X\in R^{B\times T\times C\times H\times W}$ (where B is the batch size, T is the time step length, C is the number of channels, and H × W is the spatial dimension), the dimensions are first rearranged to $X\in R^{B\times C\times T\times H\times W}$. The core of the module adopts a dual-path feature extraction mechanism, which performs mean pooling and max pooling operations on the input features in the channel dimension, as follows:

(4) $$\begin{array}{ll} F_{avg} & =\frac{1}{C}\sum_{i=1}^{C}X_{perm}^{\left(i\right)}\in R^{B\times 1\times T\times H\times W}\\ F_{max} & =\underset{i\in [1,C]}{\max}X_{perm}^{\left(i\right)}\in R^{B\times 1\times T\times H\times W} \end{array}$$

Among them, $F_{avg}$ captures global motion trends (such as hand movement trajectories) and $F_{max}$ emphasizes local salient features (such as fingertip shape changes). The two are spliced along the channel axis to form a joint representation. as follows:

(5) $$F_{concat}=\Gamma (F_{avg},F_{max})$$

The fusion feature input parameterized 3D convolution layer is expressed as follows:

(6) $$A_{raw}=W*F_{concat}$$

In the aforementioned equation, ∗ denotes a 3D convolution operation and the convolution kernel size is set to 3 × 7 × 7, covering both short-term temporal dynamics (=3 frames) and spatial local regions (=49 pixels). The attention weights are then normalized using the Sigmoid activation function, and the final output is feature-calibrated using the Hadamard product.

(7) $$Y=X⊙P^{-1}(A)\in R^{B\times T\times C\times H\times W}$$

**Figure 6:**
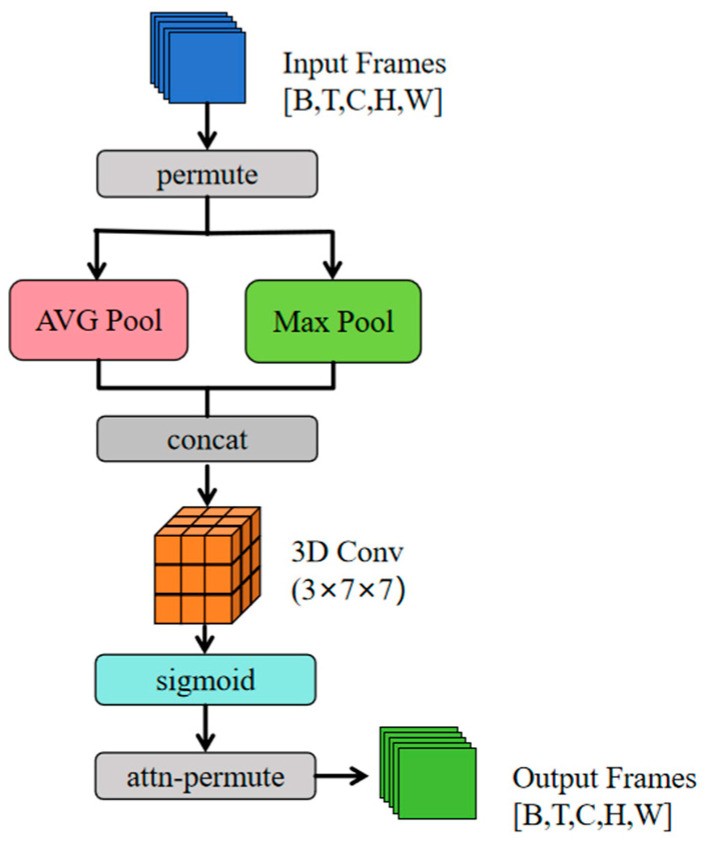
This figure shows the internal architecture of Soft Spati-Temporal Attention. Pink refers to the “AVG Pool” layer. Green refers to the “Max Pool” layer and “Output Frames”. Orange refers to the “3D Conv (3 × 7 × 7)” layer. Light blue refers to the “sigmoid” layer. Gray refers to simple operations on tensors, such as permute and contact.

From an information theory perspective, this module actually maximizes the mutual information between the input features and the sign language category labels. The mutual information of sign language videos can be decomposed into the following:

(8) $$I(S;V)=I(S;V_{T})+I(S;V_{S})-I(S;V_{T};V_{S})$$

where S is the semantic label and represents temporal dynamics and spatial structure information, respectively. Conventional separation modeling techniques neglect the incorporation of cross terms, consequently leading to the disregard of spatiotemporal collaborative characteristics. The SSTA module employs a 3D convolution kernel to explicitly model the spatiotemporal joint distribution, and its parameter optimization objective can be expressed as follows:

(9) $$\underset{W}{\min}E_{X}\left[L_{CE}\left(\Phi (Y),S\right)+\lambda \|A-M_{gt}{\|}_{F}^{2}\right]$$

where min_W denotes minimizing the objective function with respect to the model’s learnable parameters W (e.g., weights and biases in the SSTA module and classifier) and ℜ_X represents the expectation calculated over the input sign language video data X to capture its overall statistical characteristics. The cross-entropy loss, quantified by the term L_(CE)(Φ(Y), S), is the difference between the predictions of the classifier, Φ (taking processed features, Y, as input), and the true semantic label, S. Furthermore, λ·‖A − M_(gt)‖_F^2^ is a regularization term, where λ is the coefficient balancing the loss and regularization, A is the model-generated attention distribution, M_(gt) is the human cognition-aligned ground truth attention distribution, and the squared Frobenius norm ensuring A aligns with M_(gt). This information-theoretic interpretation provides a theoretical basis for the effectiveness of the module and explains why the attention mechanism can improve sign language recognition performance. Figure 6 shows the complete architecture of SSTA.

### 3.5. Transformer Encoder

Sign language video recognition faces unique challenges in temporal modeling. As a visual language, sign language relies on the precise temporal combination of continuous hand movements for semantic expression. Recurrent neural networks (RNNs) and their variants, such as Long Short-Term Memory (LSTM) networks, are inherently limited in their ability to model long sequences. As sequence length increases, the vanishing gradient problem emerges, impeding the models’ capacity to learn long-range dependencies. Unidirectional temporal modeling falls short in fully leveraging contextual information from future frames. Additionally, individual differences in sign language movement rates, such as variations in gesture speed between elderly people and young adults, necessitate that models exhibit temporal invariance. These limitations impose significant constraints on the recognition accuracy of complex composite gestures.

In order to address these challenges, the Transformer encoder is introduced as the core of temporal modeling, with the appropriate modifications [35]. The module under consideration comprises a position encoding system and multiple cascaded encoding layers, which are mathematically defined as follows:

Given the input frame feature sequence $X\in R^{T\times d}$ (T is the sequence length and d is the feature dimension), the sequence first generates position embeddings through learnable position encoding, as follows:

(10) $$P=\sigma (W_{p}\cdot t+b_{p})$$

where $t=[0,1,...,T-1{]}^{T}$ is the temporal position vector, $W_{p}\in R^{d\times 1}$ is the trainable weight matrix, and σ denotes the ReLU activation function. The position-enhanced features are represented as

(11) $$\tilde{X}=X+P$$

The design overcomes the limitations of the original Transformer’s sine encoding and adapts to capture the unique motion rhythm patterns of sign language through parameterized learning. The enhanced features are subsequently integrated into the Transformer encoding stack, with each layer comprising the following two core submodules: Multi-head Self-Attention (MSA) and Feedforward Network (FFN).

(12) $$\begin{array}{ll} Z^{\left(l\right)} & =LayerNorm\left({\tilde{X}}^{\left(l-1\right)}+MSA({\tilde{X}}^{\left(l-1\right)})\right)\\ {\tilde{X}}^{\left(l\right)} & =LayerNorm\left(Z^{\left(l\right)}+FFN(Z^{\left(l\right)})\right) \end{array}$$

Among them, the multi-head attention mechanism performs the parallel computation of individual attention heads, as follows:

(13) $$MSA(Q,K,V)=Concat({head}_{1},...,{head}_{h})W^{O}$$

Feedforward Networks enhance representation capabilities through nonlinear transformations, as follows:

(14) $$FFN(x)=ReLU(xW_{1}+b_{1})W_{2}+b_{2}$$

**Figure 7:**
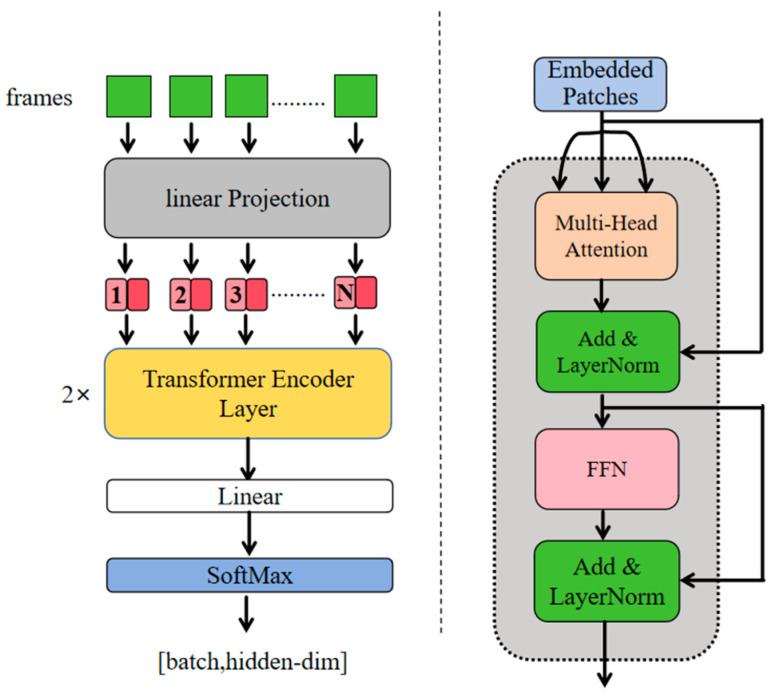
The (**left**) side of the figure illustrates the overall architecture of the Transformer encoder, while the (**right**) side delineates the architecture of the Transformer encoding layer. Gray refers to the linear Projection. Red refers to the numbered sequences. Yellow refers to the Transformer Encoder Layer. White refers to the Linear layer. Blue refers to the SoftMax layer. Light blue refers to the Embedded Patches. Orange refers to the Multi-Head Attention layer. Green refers to the “Add & LayerNorm” layers. Pink refers to the Feed-Foward Network.

Firstly, the self-attention mechanism eliminates the vanishing gradient problem through fully connected temporal modeling. Secondly, bidirectional attention weights dynamically aggregate global context, thereby enabling the model to concurrently utilize past and future frame information to identify key frames, such as emphatic pauses in sign language. In conclusion, the multi-head attention mechanism autonomously acquires temporal patterns across disparate subspaces. The focus of some researchers is on the continuity of gesture trajectories, while the focus of others is on the instantaneous changes in finger joint angles. This multi-granularity feature fusion is crucial for distinguishing similar gestures. The model utilizes a stacking approach with multiple layers of encoders to progressively refine a discriminative temporal representation $H^{\left(L\right)}\in R^{T\times d}$. This refinement provides a robust temporal semantic foundation, thereby facilitating effective classification. Figure 7 depicts our Transformer encoder:

## 4. Experimental Results and Discussion

In this experiment, to test the feasibility of the proposed framework, we deployed and trained it on the LSA64 dataset using the Ubuntu 22.04 operating system, Python version 3.10, and the open-source deep learning framework PyTorch (2.6.0). The hardware configuration included 25 vCPUs of Intel(R) Xeon(R) Platinum 8481C, with a single RTX4090 48 GB GPU used for model training. In order to assess the model’s capacity for generalization and ensure a speaker-independent evaluation, a subject-wise split was rigorously employed. This approach is critical for sign language recognition, as it tests the model’s ability to understand the linguistic content of signs rather than memorizing the specific motion patterns of individual performers. All sign language videos from two participants (Participant IDs: [005,010]) were designated as the test set, containing 640 samples (2 participants × 64 signs × 5 repetitions). The videos from the remaining eight participants constituted the training set, containing 2560 samples (8 participants × 64 signs × 5 repetitions). This split strategy effectively prevented data leakage and provided a robust evaluation of the model’s performance on unseen individuals, which is essential for real-world deployment.

### 4.1. Indicators

In sign language video recognition tasks, the performance evaluation of deep learning models needs to be combined with task characteristics (such as category imbalance, dynamic sequence feature recognition difficulty, etc.) to select appropriate indicators. The following is a detailed introduction to commonly used performance evaluation indicators:

Accuracy is the most basic indicator in model performance evaluation, used to measure the overall correct recognition ability of the model, that is, the proportion of correctly classified samples in all samples. The calculation formula is as follows:

(15) $$Accuracy=\frac{\sum_{i=1}^{C}{TP}_{i}}{N}$$

where C is the number of categories and i represents the i-th category.

Precision is defined as the proportion of samples predicted as positive that are actually positive in the model’s prediction results. The primary objective of this approach is to minimize the number of “false positives”. In the context of sign language recognition, this approach can be utilized to assess the precision of the model’s identification of particular high-frequency sign language vocabulary. The formula is as follows:

(16) $$P_{i}=\frac{{TP}_{i}}{{TP}_{i}+{FP}_{i}}$$

(17) $$Precision=\sum_{i=1}^{C}\left(w_{i}\cdot P_{i}\right)$$

$w_{i}$ is the weight of the i-th class, which is typically the proportion of samples in that class relative to the total number of samples (in this study, the weights for each class are identical).

The recall rate is defined as the proportion of all actual positive examples that are successfully identified by the model in the original sample of positive examples. The primary objective of the recall rate is to reduce the number of “false negatives”. As no samples are incorrectly predicted as non-existent categories in this experiment, the recall rate is equivalent to the accuracy rate, and, thus, it is not referenced.

The F1 score is the harmonic mean of precision and recall, used to comprehensively balance the performance of both, and is suitable for category imbalance scenarios in sign language recognition.

Since the recall rate is the same as the accuracy rate, the accuracy rate is used instead of the recall rate in actual calculations.

### 4.2. Hyperparameter Study

Conducting hyperparameter studies holds significant academic and practical value. Hyperparameters are predefined parameters set before model training, directly determining a model’s structural configuration, training process dynamics, and final performance [36,37]. Specifically, unreasonable hyperparameter configurations may lead to overfitting or underfitting of the model, while also significantly affecting training efficiency, convergence speed, and accuracy. Therefore, through systematic hyperparameter experiments, the intrinsic relationship between different hyperparameters and model performance can be revealed, providing a scientific basis for model performance optimization. Our experimental design is shown in Table 1.

As shown in the table, selecting an excessively high batch size (e.g., 16) can lead to the over-smoothing of features due to too many samples per batch. This can weaken the model’s ability to capture local patterns and reduce fitting accuracy. Conversely, an excessively low value (e.g., four) may result in excessive randomness in the samples, causing substantial gradient fluctuations and making training unstable. A value of eight strikes a balance between these extremes, ensuring sufficient feature learning while maintaining training stability.

Regarding the learning rate, an excessively high value (1 × 10^−3^) causes overly large parameter update steps, leading to oscillations that prevent convergence to the optimal solution, or even result in divergence. Conversely, an excessively low value (1 × 10^−5^) causes overly small steps, leading to slow parameter updates, insufficient model convergence, and inadequate data fitting. In contrast, 1 × 10^−4^ strikes a balance between speed and accuracy, enabling stable convergence to an optimal solution.

Concerning optimizers, SGD lacks a momentum mechanism, which results in slow convergence and susceptibility to noise, often leading to local optima. AdamW, due to its explicit weight decay (decoupled from the learning rate), may impose excessive constraints on critical motion modeling parameters (e.g., 3D convolution kernels or attention weights), yielding minimal performance differences. Adam, which combines momentum with an adaptive learning rate, achieves rapid and stable convergence, effectively adapts to data, and improves performance.

The selection of optimal parameters was guided by empirical evidence, and the final configuration included a batch size of eight, a learning rate of 1 × 10^−4^, and the Adam optimizer. This specific hyperparameter configuration achieved an optimal model performance.

After determining the hyperparameters, this study conducted a stability test and analysis of the model. The model was optimized to minimize the cross-entropy loss between the predictions and the ground truth labels. The training process was monitored using this loss value on both the training and validation sets. The mathematical expression for the cross-entropy loss for a single sample is as follows:

$$L=-\sum_{i=1}^{C}y_{i}\cdot \log({\hat{y}}_{i})$$

where *C* is the number of classes (64 for LSA64). $y_{i}$ is the true label (a one-hot encoded vector). $\log({\hat{y}}_{i})$ is the predicted probability for class *i* (the output of the final softmax layer in our network).

**Figure 8:**
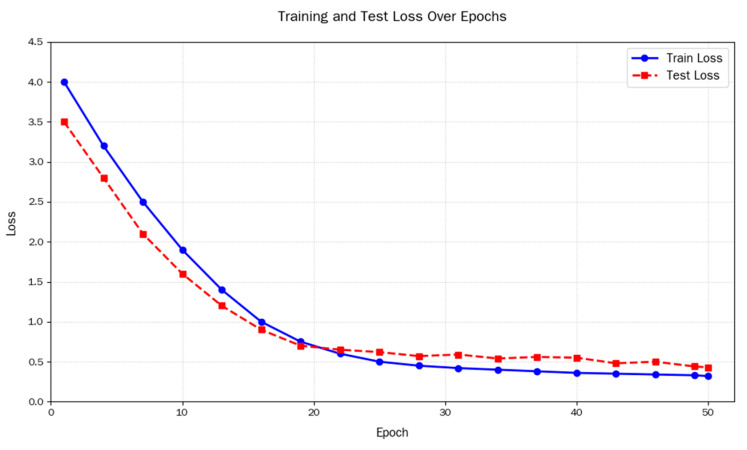
Line graph showing changes in training and testing losses under optimal hyperparameter settings.

It can be seen in Figure 8 that under the current hyperparameters, the training and test loss curves tended to stabilize at around 25 epochs. The model achieved a high training accuracy of 99.8%, while the final test accuracy remained at 96.25%, demonstrating a strong learning capacity without severe overfitting to the training data. The fluctuation range of the network was within a controllable range, and the model was relatively stable.

### 4.3. Ablation Study

Conducting ablation experiments constitutes a pivotal element in the analysis of the performance of deep learning models and the subsequent optimization of their structural design. In the context of complex model construction, which frequently incorporates multiple design modules, the presence of redundant structures or synergistic failures is a possibility [37]. By comparing the performance changes of different modules, this study can quantitatively assess the impact of modules on overall performance. The experimental design and results of the present study are displayed in Table 2.

The experimental results presented above demonstrate that ResNet shows high competence in feature extraction, and its residual connection mechanism effectively mitigates the gradient vanishing problem prevalent in deep networks. This enables the extraction of more robust and discriminative features. Simultaneously, the Transformer’s advanced global modeling capacity facilitates enhanced extraction of global temporal characteristics. Importantly, the introduction of the SSTA module enables the effective modeling of local spatiotemporal features at a minimal parameter cost, complementing the Transformer’s global modeling capability. Together, these components form a recognition framework that captures both fine-grained gesture changes and comprehensive overall semantic information. This framework provides a highly efficient and accurate solution for sign language video recognition tasks.

### 4.4. Comparison Study

The comparative models were selected based on their established relevance to video-based sign language recognition and action classification tasks, as evidenced by the recent literature. InceptionV3GRU [36] was chosen as a hybrid CNN-GRU baseline, commonly applied to sequential sign language data on datasets like LSA64. X3D-M [40] represents an efficient 3D CNN architecture optimized for video recognition, providing a lightweight yet effective benchmark. TEA [41] incorporates temporal excitation and aggregation mechanisms, making it ideal for comparing spatiotemporal modeling in action recognition. ResNet+LSTM [29] is a widely used combination for Argentine Sign Language, allowing for direct evaluation against a ResNet-based temporal model. Finally, 3D CNN+Bi-RNN [25] was included for its bidirectional RNN integration with 3D convolutions, which excels in capturing dynamic gesture sequences. This diverse set ensures a comprehensive assessment of our SSTA-ResT framework’s improvements in accuracy, precision, and efficiency. In order to facilitate a more profound comprehension of the performance and effectiveness of the proposed model in comparison to other studies, a series of comparison experiments was designed. In these experiments, the performance of other models proposed by different sign language recognition studies was compared. To ensure fairness, all comparative models underwent the same hyperparameter tuning process, as detailed in Section 4.2 (e.g., exploring batch sizes of 4–16, learning rates of 1 × 10^−5^ to 1 × 10^−3^, and optimizers including Adam, AdamW, and SGD), with the results in Table 3 reflecting the best-performing configurations for each model under identical data preprocessing and training conditions. The experimental results demonstrated that the ResNet+ssTA+Transformer method proposed in this paper attained an optimal performance in all evaluation metrics.

### 4.5. Hardware Requirements

The proposed model was evaluated for its computational efficiency on an NVIDIA RTX 4090 GPU, where the average inference time for a single sample was approximately 0.25 s, with an average memory usage of 3.91 GB. These metrics indicate efficient operation in standard computing environments. For deployment on resource-limited devices, such as mobile platforms, we estimated performance based on hardware scaling factors. On a high-end mobile GPU like the Qualcomm Adreno 750 (found in Snapdragon 8 Gen 3 devices), inference time per sample was projected to be in the range of 3–10 s, with similar memory requirements potentially reducible via optimizations. This performance level is acceptable and holds potential for non-synchronous real-time recognition tasks (e.g., applications where results within a few seconds are sufficient, similar to translation software, rather than instantaneous processing like YOLO models), such as offline data processing or periodic monitoring, where techniques like model quantization, pruning, and edge-specific frameworks (e.g., TensorFlow Lite) can further enhance suitability.

## 5. Conclusions

The proposed framework, called SSTA-ResT, represents a novel integration of ResNet, soft spatiotemporal attention (SSTA), and Transformer encoders, achieving substantial results on the LSA64 dataset. The study’s findings indicate that this approach achieved a recognition accuracy of 96.25%, a precision of 97.18%, and an F1 score of 0.9671, surpassing all other comparison methods. Notably, the SSTA module designed in this paper effectively models local spatiotemporal features with few parameters, demonstrating the significant potential of lightweight attention mechanisms in video understanding tasks. Through systematic ablation studies, the necessity of each component in the framework was verified. ResNet provides powerful spatial feature extraction capabilities, SSTA enhances local spatiotemporal correlations, and the Transformer effectively captures long-range temporal dependencies. This hierarchical feature extraction strategy enhances recognition performance while maintaining a compact model size (11.66 M parameters), thus offering a viable solution for implementing sign language recognition systems on resource-constrained devices.

The high recognition accuracy achieved in this study demonstrates the effectiveness of deep learning-based methods in reliably recognizing isolated sign language vocabulary. This finding establishes a solid foundation for developing practical applications, including sign language dictionary lookup and sign language learning aids. Accurate word-level recognition is a prerequisite for sentence-level and conversation-level sign language translation. The findings of this study will contribute to advancing sign language recognition technology from laboratory settings to practical, real-world applications.

Despite these achievements, this study has limitations that need to be addressed in future work. First, the study was conducted only on the LSA64 Argentine Sign Language dataset, which is relatively small (64 vocabulary categories) and was recorded in ideal conditions (white background with fluorescent gloves), differing from real-life scenarios. While the current study used a lab-collected dataset, we incorporated noise and augmentation to preliminarily assess robustness. Future work will include the following:

1. Real-world deployment trials and continuous sign language recognition under unconstrained conditions.
2. The current method focuses solely on word-level recognition and has not yet addressed the segmentation and recognition of continuous sign language sentences.
3. The model’s real-time performance requires further optimization to meet the low-latency requirements of actual interactive scenarios.
4. Exploring and enhancing the model’s generalization ability under different recording conditions (including camera distance and preferred hand differences).

Future research directions include enhancing the model’s generalization capabilities, exploring methods to extend recognition from word-level to sentence-level sign language, further reducing computational costs, and studying cross-language recognition and translation issues [42]. This study believes that as these issues are gradually addressed, sign language recognition technology will truly become an important tool for the deaf community to integrate into digital society.

## Figures and Tables

**Table 1:** Hyperparameter comparison study.

Batch Size	Lr	Optimizer	Accuracy	Precision	F1-Score
8	1 × 10^−4^	Adam	96.25%	97.18%	0.9671
16	1 × 10^−4^	Adam	90.88%	94.65%	0.9269
4	1 × 10^−4^	Adam	88.19%	90.87%	0.8952
8	1 × 10^−5^	Adam	63.82%	65.81%	0.6479
8	1 × 10^−3^	SGD	1.56%	9.42%	0.0268
8	1 × 10^−4^	AdamW	93.98%	95.76%	0.9486

**Table 2:** Ablation study.

Method	Backbone	Neck	Classifier	Accuracy	Precision	F1-Score	Parames
SSTA-ResT(ours)	ResNet	SSTA	Transformer	96.25%	97.18%	0.9671	11.66 M
Ablation1	ResNet	-	Transformer	91.78%	95.57%	0.9353	11.66 M
Ablation2	ResNet	SSTA	-	67.72%	67.28%	0.6750	11.30 M
Ablation3	ResNet	SE [38]	Transformer	89.52%	91.24%	0.9037	11.70 M
Ablation4	ResNet	CA [39]	Transformer	89.76%	92.09%	0.9091	11.69 M
Ablation5	MobileNet	SSTA	Transformer	86.03%	87.32%	0.8669	4.05 M
Ablation6	CNN	SSTA	Transformer	87.11%	88.76%	0.8792	4.73 M

**Table 3:** Comparison study.

Method	Accuracy	Precision	F1-Score	Params
InceptionV3-GRU [36]	77.25%	77.61%	0.7753	35 M
X3D-M [40]	86.58%	88.13%	0.8729	23.2 M
TEA [41]	85.79%	88.22%	0.8698	21.9 M
ResNet+LSTM [29]	87.98%	89.95%	0.8892	4.73 M
3D CNN+Bi-RNN [25]	91.54%	93.01%	0.9235	15.13 M
SSTA-ResT(ours)	96.25%	97.18%	0.9671	11.66 M

## Data Availability

The experimental data are available at https://facundoq.github.io/datasets/lsa64/ (accessed on 30 July 2025).
